# Wild Boars as an Indicator of Environmental Spread of ESβL-Producing *Escherichia coli*

**DOI:** 10.3389/fmicb.2022.838383

**Published:** 2022-04-01

**Authors:** Alessandra Mercato, Claudia Cortimiglia, Aseel Abualsha’ar, Aurora Piazza, Federica Marchesini, Giovanni Milani, Silvia Bonardi, Pier Sandro Cocconcelli, Roberta Migliavacca

**Affiliations:** ^1^Department of Clinical-Surgical, Diagnostic and Pediatric Sciences, Unit of Microbiology and Clinical Microbiology, University of Pavia, Pavia, Italy; ^2^Department for Sustainable Food Processes, Università Cattolica del Sacro Cuore, Piacenza, Italy; ^3^Department of Veterinary Science, Unit of Food Inspection, University of Parma, Parma, Italy

**Keywords:** wild boars, Italy, ESβLs-producing *Escherichia coli*, ST131, ST10

## Abstract

Antimicrobial resistance (AMR) represents an increasing issue worldwide, spreading not only in humans and farmed animals but also in wildlife. One of the most relevant problems is represented by Extended-Spectrum Beta-Lactamases (ESβLs) producing *Escherichia coli* because they are the cause of important infections in human. Wild boars (*Sus scrofa*) as a source of ESβLs attracted attention due to their increasing density and their habits that lead them to be at the human-livestock-wildlife interface. The aim of this study was to increase the knowledge about the ESβLs *E. coli* strains carried by wild boars living in a particularly high-density area of Northern Italy. The analysis of 60 animals allowed to isolate 16 ESβL-producing *E. coli* strains (prevalence 23.3%), which were characterised from a phenotypical and molecular point of view. The overall analysis revealed that the 16 isolates were all not only ESβL producers but also multidrug resistant and carried different types of plasmid replicons. The genome analysis performed on a subset of isolates confirmed the heterogeneity observed with pulsed-field gel electrophoresis (PFGE) and highlighted the presence of two pandemic sequence types, ST131 and ST10, with different collections of virulence factors. The genomic context of ESβL genes further evidenced that all of them were surrounded by transposons and insertion sequences, suggesting the possibility to exchange AMR genes. Overall, this study shows the worrying dissemination of ESβL-producing *E. coli* in wild boars in Northern Italy, suggesting the role of these animals as a spreader of AMR and their inclusion in surveillance programmes, to shed light on the “One Health” complex interactions.

## Introduction

The concept of One Health is based on an existing connection between humans, animals, plants, and environmental health (Karesh et al., 2012). In recent years, this concept has been reformulated underlying the role of interconnected ecosystems, i.e., geographically closed systems, in the occurrence of common health traits (van Bruggen et al., 2019). Such interactions between the different domains are supported by the exchange of microbial communities between humans, animals, plants, and the local environment, thus influencing the health conditions of organisms, communities, and ecosystems (van Bruggen et al., 2019). In this context, despite the importance of microbial communities’ distribution across domains, antimicrobial resistance (AMR) is considered the quintessential One Health issue (Robinson et al., 2016).

The emergence and distribution of antimicrobial-resistant bacteria (AMB) between people and animals have been often attributed to abuse/misuse of antimicrobials in husbandry practices (sub-therapeutical doses and long-lasting treatments), which create the ideal conditions for bacteria to develop resistance (Robinson et al., 2016). AMB shed by farmed animals may contaminate agricultural areas through the spreading of manure and reach surface water, thus contributing to contamination of soil, plants, and wild animals (Laxminarayan et al., 2013). In contrast, important sources of AMR bacteria are represented by hospitals, which release their sewage drains and wastewaters in the environment and represent a menace for different ecosystems (Baquero et al., 2008). Even if hospital effluents are treated in wastewater treatment plants (WWTPs), AMR bacteria and their resistant genes can survive (Yang et al., 2016), thus persisting in the environment and circulating in the ecosystems.

It is commonly assumed that this anthropogenically derived pollution of antimicrobials, AMB, and resistance genes from human waste and livestock farms is the source of wildlife contamination (Carroll et al., 2015). As a matter of fact, wildlife commonly carries AMB deriving from contact with anthropogenic sources that pollute the environment with AMB and/or with antimicrobials (Bondo et al., 2016; Furness et al., 2017; Swift et al., 2019). Nevertheless, despite the isolation of AMB, some studies suggest a lack of evidence of direct contact of wild animals with human or livestock sewage, slurry, or faeces, thus questioning clear pathways of AMR transmission (Furness et al., 2017).

Most of the studies in wild animals are focused on the detection of *Escherichia coli* producing Extended-Spectrum Beta-Lactamases (ESβLs), which are known to be emerging in livestock as well as in wildlife, thus escaping from human clinical settings (Guenther et al., 2011; Atterby et al., 2017; Bonardi et al., 2018; Zendri et al., 2020). It has been reported that the occurrence of ESβL-producing *E. coli* in wild animals, especially wild birds, could be a spill-over form of environmental pollution from human and livestock sources (Guenther et al., 2011). As a matter of fact, wild birds have been considered environmental indicators, reservoirs, and even spreaders of AMR (Bonnedahl and Järhult, 2014). However, the role of AMR indicators could also be postulated for wild mammals like wild boars (*Sus scrofa*).

During the last decades, wild boars have been expanding in Europe despite them being one of the most intensively hunted ungulate species (European Food Safety Authority (EFSA), 2014). Due to their high reproductivity rate and omnivorous habits, their population is likely to an overgrowth in many territories (Keuling et al., 2013; European Food Safety Authority (EFSA), 2014). In Italy, wild boars are among the most common wild ungulates, with densities varying from 0.01–0.05 to 2.32–10.5 animals/km^2^ across the whole Italian territory (Pittiglio et al., 2018). In the region of the study (Emilia-Romagna region; 22,451 km^2^), a density of 1.37–2.31 wild boars/km^2^ was estimated, thus consisting of a regional wild boar population ranging between thirty and fifty thousand animals. Since these animals are also used for the production of non-thermally treated foods, such as cured meats and dry fermented sausages, it is possible for bacteria from animals to reach the consumers *via* these foods.

The main aims of the study were the evaluation of (i) prevalence of ESβL-producing *E. coli* among a small group of wild boars hunted in Northern Italy (Emilia-Romagna region); (ii) molecular typing of the isolates to characterise AMR determinants, virulence determinants, and phylogenetic groups; and (iii) comparison with strains isolated from different sources, including human, food, wild, and companion animals.

## Materials and Methods

### Sample Collection and Isolation of ESβL-Producing *Escherichia coli* Strains

From June to December 2018, 60 wild boars (35 females and 25 males) that were hunted in Parma Province, Northern Italy, were tested for the presence of ESβL-producing *E. coli*. Of them, 14 animals (23.3%) belonged to age class 0 (young; <12 months), 17 (28.3%) to age class 1 (subadults; 13–24 months) and 29 (48.3%) to age class 2 (>24 months). For this survey, only animals dead since less than 5 h were selected. Mesenteric lymph nodes (MLNs) and faecal samples were collected immediately after evisceration. MLNs were washed with sterile saline solution and decontaminated using ethyl alcohol before being placed in sterile containers. Faecal samples were collected from the colon and placed in sterile containers. The samples were transported to the laboratory at refrigerated conditions and tested the day of collection.

Mesenteric lymph nodes were cut into small pieces (0.1–0.2 cm) by using sterile scissors. Notably, 5–10 g of MLNs, according to their size, and 10 g of faeces were diluted 1:10 in buffered peptone water (BPW; Oxoid, Basingstoke, United Kingdom) and incubated at 37 ± 1°C for 18–20 h. A 100 μl loopful of the cultures were streaked onto MacConkey (Oxoid) agar plates added with a disk containing cefotaxime (CTX; 5 μg) and incubated at 44 ± 1°C for 21 ± 3 h. Colonies grown in the proximity of the antimicrobial disk were selected to be seeded onto Tryptone Soya Agar (TSA, Oxoid) and Tryptone Soya Broth (TSB, Oxoid) and incubated at 44 ± 1°C for 21 ± 3 h. Indole production was tested by adding James’ reagent to TSB cultures. From TSA plates of indole-positive cultures, one well-isolated colony was identified at the species level with the Microgen^®^ GN-A (Biogenetics, Padua, Italy) system. *E. coli* isolates were analysed using the Kirby-Bauer test following EUCAST recommendations (2018) using disks produced by Oxoid containing CTX (5 μg), ceftazidime (CAZ; 10 μg). *E. coli* ATCC 25922 was used as a quality control microorganism. The strains which showed an inhibition zone diameter <17 mm for CTX and <19 mm for CAZ were selected for ESβL testing. Phenotypic identification of ESβL-producing *E. coli* was performed using the ESβL-Confirm Kit (Rosco Diagnostica, Taastrup, Denmark) following the manufacturer’s instructions.

### Antimicrobial Susceptibility Test

The antimicrobial susceptibility profiles for the phenotypically identified ESβL-producing *E. coli* isolates were determined using MicroScan Gram-negative MIC/Combo panels and analysed through the semi-automated system MicroScan autoSCAN-4 (Beckman Coulter) following the manufacturer’s instructions. Clinical categorisation of the isolates was performed based on the EUCAST 2019 clinical breakpoints.^1^

### Molecular Investigation of Resistance Determinants

The presence of ESβL and carbapenemases determinants was investigated by microarray Check-Points CT 103 XL Check-MDR assay (Check-Points Health B.V., Wageningen, Netherlands) and PCR. To determine the exact allelic variant of *bla*_*CTX–M*_, two-directional DNA sequencing was performed. PCR amplicons of 593 bp, obtained using the primer pair Fw: 5′-ATGTGCAGYACCAGTAARGT-3 and Rev: 5′-TGGGTRAARTARGTSACCAGA-3′, were purified using a Wizard^®^ SV Gel and PCR Clean-Up System (Promega, Madison, WI, United States). DNA sequencing was performed using the Microsynth services (Microsynth Seqlab, Germany). The alignment between the forward, reverse, and reference DNA sequences was accomplished using ChromasPro software (Technelysium Pty Ltd, South Brisbane, Australia) and analysed using the BLAST software.^2^ The PCR assays were performed to assess the presence of resistance determinants against quinolones, namely *qnrB*, *qnrS*, and *aac(6′)-Ib-cr*, and aminoglycosides, like *aadA*, *armA*, *rmtB*, and *rmtC*.

### Conjugation Assay and Plasmid Typing

To assess the possible transferability of the *bla*_*CTX–M*_ determinants identified, conjugation assays in liquid were performed using three different *E. coli* strains as recipients, including the *E. coli* K12 strain J62 (pro-, his-, trp-, lac-, and Sm-R), J53-2 (met-, pro-, and rif-R), and J53 AzideR. Donor strains in the logarithmic growth phase were mixed with recipients in the early stationary phase in a 1:10 ratio in Mueller Hinton broth (Oxoid, Basingstoke, UK), and the mixture was incubated at 37°C overnight. The transconjugants were selected on MacConkey agar containing cefotaxime (2 mg/L) plus streptomycin (1,000 mg/L), rifampicin (100 mg/L), or sodium azide (100 mg/L) (Tartor et al., 2021). The detection sensitivity of the assay was approximately from eight to ten transconjugants per recipient. At least three possible transconjugant colonies for each recipient were subjected to antimicrobial susceptibility testing and PCR to confirm *bla*_*CTX–M*_ gene presence using the primers mentioned above. Plasmids were also typed for both the donors and the transconjugants based on their incompatibility groups using the PCR-based replicon typing scheme PBRT 2.0 Kit (Diatheva, Fano, Italy). Moreover, random amplified polymorphic DNA (RAPD)-PCR was performed for the donor, transconjugants, and recipients using the RAPD-PCR Kit (Amersham BioSciences UK Limited, England) to definitely distinguish transconjugants from donor strains.

### Pulsed-Field Gel Electrophoresis

The pulsed-field gel electrophoresis (PFGE) was performed using the *Xba*I restriction enzyme, and the obtained genomic fragments were separated on a CHEF-DR II apparatus (Bio-Rad, Milan, Italy) for 22 h at 14°C. Bacteriophage λ concatenamers were used as DNA size markers. DNA restriction patterns of scanned gel pictures were interpreted following cluster analysis using the Fingerprinting II version 3.0 software (Bio-Rad) using the unweighted pair-group method with arithmetic averages (UPGMA). Only bands larger than 48 kb were considered for the analysis. The Dice correlation coefficient was used with a 1.0% position tolerance to analyse the similarities of the banding patterns, and a similarity threshold of 90% to define clusters. The restriction patterns of the genomic DNA from the isolates were analysed and interpreted according to the criteria of Tenover et al. (1995).

### Phylogenetic Group Investigation

Phylogenetic group typing was performed for the 16 ESβL-producing *E. coli* isolates according to Clermont et al. using PCR assays targetting the genes *chuA*, *yjaA*, the DNA fragment TSPE4.C, and *arpA* gene as described previously (Clermont et al., 2013).

### Whole-Genome Sequencing and *in silico* Analysis

**FIGURE 1:**
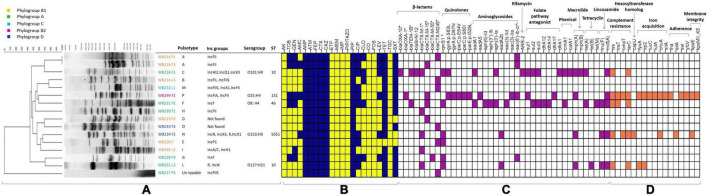
The figure includes all the phenotypical and molecular characterisation, including the analysis performed with whole-genome sequencing (WGS). The part **(A)** is related to the molecular typing by pulsed-field gel electrophoresis (PFGE), with the identification of pulsotypes, phylogroups, and plasmid replicons, for all the extended-spectrum beta-lactamase (ESβL)-producing *Escherichia coli* strains, while STs and serogroups are evidenced for the sequenced strains. The heat map **(B)** shows the phenotypical resistance profile of all ESβL-producing *E. coli*, where yellow identifies susceptible phenotype, while blue identifies the resistance phenotype. The molecular determinants found by PCR and genome analysis are evidenced in the heat map in purple **(C)**, while the virulence genes harboured by the sequenced strains are shown in orange **(D)**. AMR genes and virulence factors are grouped by evidenced categories. AMC, amoxicillin/clavulanic acid; AMP, ampicillin; PIP, piperacillin; ATM, aztreonam; FEP, cefepime; CTX, cefotaxime; CAZ, ceftazidime; CXM, cefuroxime; CIP, ciprofloxacin; LEV, levofloxacin; TZP, piperacillin-tazobactam, AK, Amikacin; GM, gentamicin; TOB, tobramycin; CLO, chloramphenicol; ERT, ertapenem; IMI, imipenem; MEM, meropenem; TET, tetracycline; TMS, trimethoprim/sulfamethoxazole. *Determinants detected by PCR.

Notably, five out of the 16 ESβL-producing *E. coli* strains were selected taking into consideration the highest level of antibiotic resistance and different phylogroups and subjected to whole-genome sequencing (WGS) for further investigation (Figure 1). The total DNA was extracted from pure overnight culture using the E.Z.N.A.^®^ Bacterial DNA Kit (Omega Bio-Tek, Norcross, GA, United States), following the manufacturer’s instructions. The DNA concentration was determined using the Qubit 2.0 Fluorometer (Thermo Fisher Scientific) and visualised on 0.8% agarose gel to check the DNA integrity. DNA was sequenced by Fasteris (Geneva, Switzerland) using the Illumina Miseq platform (Illumina Inc., San Diego, CA, United States) with 300 paired-end runs.

The quality of raw reads was evaluated using FastQC software. SPAdes tool on the PATRIC website was used to perform the *de novo* assembly, discarding contigs with a length below 400 bp (Wattam et al., 2017), and contigs were annotated using Prokka version 1.13.3 with *e*-value cut-off default (Seemann, 2014). *In silico* investigation of Multilocus Sequence Type (MLST), serotype, fimH subtyping, and phylogroup was investigated using MLST 2.0 for *E. coli* #1 (Larsen et al., 2012), SerotypeFinder 2.0 (Joensen et al., 2015), FimTyper-1.0 (Roer et al., 2017), and default threshold. ResFinder 4.1 (Bortolaia et al., 2020), PlasmidFinder 2.1 (Carattoli and Hasman, 2020), and VirulenceFinder 2.0 (Malberg Tetzschner et al., 2020) were used to detect AMR genes, plasmid replicons, and virulence factors, respectively.

Contigs harbouring ESβL determinants were annotated using Prokka, and the genetic environment of AMR genes was investigated using Geneious Prime version 2021.2.2 and Isfinder (Siguier et al., 2006). In particular, since *bla*_*CTX–*15_ is known to be flanked by the transposon element ISEcp1 (AJ242809), a BLASTn search was performed on *bla*_*CTX–*15_-positive genomes.

The phylogeny among wild boar strains was investigated analysing the pan-genome using Roary (Page et al., 2015). Then, the phylogenetic relationship between ST131 and ST10 strains from wild boars and other strains with the same sequence type (ST) and isolated from different sources was investigated.

We explored the genomes deposited in PATRIC and EnteroBase (Zhou et al., 2020), for which information about the ST was available. The data about ST were confirmed using MLST 2.0 for *E. coli* #1 (Larsen et al., 2012). Moreover, metadata about isolation sources (human, animal, and food), isolation country, host health (where applicable), and ESβL genes were also considered. All the selected genomes were re-analysed with ABRicate using the ResFinder database^3^ and typed through SerotypeFinder.

Fifty-seven ST131 ESβL-producing *E. coli* genomes with the mentioned features were obtained from PATRIC and EnteroBase (Supplementary Table 1). When fastQ files were available, they were assembled with Spades. FASTA files were re-annotated using Prokka version 1.13.3 with *e*-value cut-off default (Seemann, 2014). The GFF files of all the downloaded ST131 and of WB 249 F2 strain were used for the pan and core genome analysis using Roary (Page et al., 2015). The Newick file resulting from Roary analysis was uploaded in iTOL for viewing the relationship based on single nucleotide polymorphism (SNP) detected in the core genome.

The same approach was adopted for the comparison between ST10 wild boar strains and genome sequences of ST10 strains isolated from different sources (Supplementary Table 2). Genome sequences of ST10 EsβL-producing *E. coli* were retrieved from the PATRIC database. Sequences were screened confirming the ST group, and a subset of 49 genomes was selected, considering the source (human, companion, and wild animal food) and the positivity for ESβL genes.

## Results

### Wild Boars Are Carriers of ESβL-Producing and Multidrug Resistant *Escherichia coli*

Notably, 14 wild boars out of 60 (23.3%; 95% CI 14.4–35.4) were found to be positive for the presence of CTX- and CAZ-resistant *E. coli*. A total of 16 *E. coli* isolates were collected from the 14 animals from MLN (5/60; 8.3%) and faeces (11/60; 18.3%) samples, with three wild boars positive both in MLNs and faeces. Positive wild boars were represented by five females (35.7%) and nine males (64.3%) belonging to age class 0 (4/14; 26.6%) and age class 2 (10/29; 34.5%). All the 16 isolates were positive to the ESβL-Confirm Kit.

A wider evaluation of antimicrobial susceptibility profiles of the 16 ESβL-producing *E. coli* showed 100% non-susceptibility to penicillins, third-generation cephalosporins (3GCs) and fourth-generation cephalosporins (4GCs), tetracyclines, monobactams, and tetracycline; 75% to trimethoprim/sulfamethoxazole, 37.5% to chloramphenicol, among fluoroquinolones, 62.5% to ciprofloxacin and 31.25% to levofloxacin, and against aminoglycosides, 31.25% to tobramycin and 37.5% to gentamycin, and 18.75% to amoxicillin/clavulanate. All isolates resulted fully susceptible to carbapenems, amikacin, tigecycline, fosfomycin, piperacillin/tazobactam, and colistin (Figure 1).

Genotypic investigation was then used to determine the identity of genes conferring the phenotypic resistance. The presence of the bla_*CTX–M*_-type genes was detected by microarray and confirmed using targetted PCR. bla_*CTX*–M–245_, bla_*CTX–M–*15_, and bla_*CTX–M–*1_, were found in 31.3% (*n* = 5/16), 25% (*n* = 4/16), 25% (*n* = 4/16) strains, respectively. Among ESβL-producing strains, fluoroquinolone resistant isolates (56.3%, *n* = 9/16) harboured either qnrS (55.6%, *n* = 5/9), aac(6′)Ibcr (33.3%, *n* = 3/9), or both qnrS and aac(6′)Ibcr (1.1%, *n* = 1/9) genes, while aminoglycoside resistant isolates (37.5%, *n* = 6/16) harboured either aadA (33.3%, *n* = 2/6), aac(6′)Ibcr (50%, *n* = 3/6), or aadA and aac(6′)Ibcr (16.7%, 1/6) determinants (Figure 1).

### Plasmid Profile and Conjugation Results

The possible presence of plasmids was assessed for all 16 *E. coli* isolates. IncFII was the prevalent incompatibility group found in the isolates (*n* = 6/16). Other groups detected included IncN (*n* = 2/16), IncX1 (*n* = 2/16), IncX3 (*n* = 2/16), IncFIIS (*n* = 2/16), IncFIIK (*n* = 1/16), IncF (2/16), IncFIA (*n* = 1/16), IncA/C (*n* = 1/156), IncH12 (*n* = 1/16), IncH1 (*n* = 1/16), IncQ1 (1/16), and IncP1 (*n* = 1/16). Two isolates resulted negative for all the incompatibility groups searched.

All strains were then subjected to conjugation experiments, and a lateral transfer of resistance genes was observed in 25% (n = 4/16) of them. The resistance profiles of donors and transconjugants confirmed the lateral transfer of 3GC resistance. PCR analysis confirmed the presence of a bla_*CTX–M*_-type gene in all the transconjugants. Inc group plasmid and RAPD profile analysis performed on all the transconjugants confirmed that the bla_*CTX–M*_ gene was plasmid-encoded and that plasmids are mobilised by conjugation to the *E. coli* J53 or *E. coli* J62 recipient.

### Molecular Typing Reveals High Heterogeneity Among ESβL-Producing *Escherichia coli*

The PFGE analysis of the 16 ESβL-producing *E. coli* isolates showed a high clonal heterogeneity, revealing 14 different pulsotypes, even for the isolates recovered from MLNs and faeces of the same wild boar (Figure 1). One *E. coli* isolate resulted not typable with this method.

The assignment to *E. coli* phylogenetic groups of the 16 isolates revealed that the 37.5% (*n* = 6/16), all CTX-M-producers, belonged to phylogroup A; 37.5% (*n* = 6/16) belonged to phylogroup B1; 12.5% (*n* = 2/16) to phylogroup D; 6% (*n* = 1/16) to phylogroup B1, and 6.25% (*n* = 1/16) to phylogroup C (Figure 1).

### In-Depth Genomic Characterisation: Typing, Virulence, Antimicrobial Resistance, and Dissemination Potential

Among the 16 ESβL-producing *E. coli* strains, five of them were selected considering the resistance to a high number of antimicrobials and the phylogroup and sequenced for further deep characterisation. As shown in Table 1, the *in silico* analysis evidenced four different STs, and in particular, two strains with ST10, one strain with ST131, one strain with ST46, and one strain with ST5051. The ST10 strains belonged to two different serogroups (i.e., O101:H9 and O127:H21), and the ST131 strain belonged to O25:H4 serogroup; O8:H4 and O153:H9 were detected as serogroup of ST46 and ST505, respectively. While the ST131 strain showed the fimbrial variant fimH30, the other isolates harboured fimH54, except for WB221F2, which carries fimH34.

**TABLE 1:** Main genome features of five extended-spectrum beta-lactamase (ESβL)-producing *Escherichia coli* selected strains isolated from wild boars.

	WB218 F1	WB221 F2	WB231 L2	WB234 F2	WB249 F2
Genome size (bp)	4917558	4790178	4649545	5051588	5070855
N°contigs	113	164	74	78	73
MLST	10	46	10	5051	131
FimH group	54	34	54	54	30
Serogroup	O101: H9	O8: H4	O127:H21	O153:H9	O25:H4

The analysis of the genome led to a deep characterisation of virulence and AMR content. The analysis of the virulence profile evidenced the absence of Shiga-toxin genes (*stx1* and *stx2*) and intimin gene (*eae*). Some other putative virulence factors were identified. In particular, WB249F2 shows a large collection of genes involved in adherence (*iha* and *yfcV*), complement resistance (*iss*, *traT*, and *ompT*), iron acquisition (*iucC*, *iutA*, *chuA*, *fyuA*, and *irp2*), and membrane integrity (*kpsE* and *kpsMII_K5*). The other strains showed a less enriched panel of virulence factors, some of whom were in common with the WB249F2 strain. Differently from all other strains, WB234F2 carried hexosyltransferase homologue gene (*capU*) and Salmonella hilA homologue (*eilA*).

A more accurate and comprehensive typing of antimicrobial determinants was assessed using the WGS analysis (Figure 1). Among ESβL genes, the five sequenced wild boar strains were confirmed to carry the same determinants found by molecular analysis and, moreover, were able to detect other genes. In fact, the screening of the genome revealed that the WB218F1 strain was also a carrier of *bla*_TEM–1B_ and *bla*_SHV–12_ genes. It is interesting to notice that while all strains were positive for *bla*_*CTX–M*_ genes, the WB218F1 also carried all the other EsβL determinants.

Genome analysis of other resistance genes confirmed the most resistance phenotypes against other antimicrobials, identifying determinants directly related to the phenotype. In fact, WB218F1 showed a point mutation in the *gyrA* gene (S83L) linked to the fluoroquinolone phenotype, while the resistance of the WB249F2 strain was due to mutations in *gyrA* (S83L and D87N) gene associated with the *parC* (E84V, S80I) and *parE* (I529L) mutations.

WB218F1 and WB221F2 were positive for the *cmlA1* gene, responsible for the resistance against chloramphenicol together with *catA1* and *flor1*, respectively. The analysis revealed that strains (WB221F2 and WB249F2) phenotypically resistant to aminoglycosides such as gentamicin and tobramycin carried not only *aac(6′)-Ib-cr* genes but also other genes belonging to the aminoglycoside-(3)-*N*-acetyl-transferase families, such as *aac(3)-IId* and *aac(3)-IIa*. From a phenotypic point of view, all five strains resulted in resistance to tetracycline; this evidence was confirmed at the genome level only for three strains, which carried *tet(A)*, *tet(B)*, and *tet(M)*. The tetracycline resistance for WB234F2 was related to the presence of *mdf(A)*, a multidrug-resistant (MDR) determinant, while in the case of the WB249F2 strain, no genes were identified. Moreover, the analysis lead to identify the presence of other genes linked to the resistance to trimethoprim-sulfamethoxazole such as *dfrA1*, *dfrA12*, *dfrA17*, *sul3*, *sul2*, and *sul1* in WB218F1, WB221F2, and WB249F2 genomes.

No gene mutations usually linked to levofloxacin were identified, due to the absence of correlation in the ResFinder/PointFinder database (Mahfouz et al., 2020).

The possibility of AMR genes to be mobilised between strains is directly linked to their localisation on mobile genetic elements, i.e., plasmids and transposons. The genome of five strains was screened for plasmid replicons; moreover, this information was linked to the investigation of genome makeup around AMR genes in order to study the possibility of gene sharing through mobile elements.

The analysis with PlasmidFinder matched with the results of PCR (Table 2).

**TABLE 2:** Identification of plasmid replicons in the sequenced ESβL-producing *Escherichia coli* strains.

Strain	Plasmid replicon	Contig position	Contig length (bp)
WB218 F1	IncHI2	46	10,181
	IncHI2 2	42	22,264
	IncX3	37	35,167
WB221 F2	IncFIB	92	4,852
		12	104,785
WB231 L2	IncN	26	31,042
	IncR	32	15,115
WB234 F2	IncN	28	31,174
	IncR	33	11,243
	IncX1	25	45,110
WB249 F2	IncFIA	29	17,426
	IncFII	35	8,144

*For each strain, the incompatibility group (plasmid replicon) is highlighted with the related number of contig on which the replicon was found and the length of the contig.*

**FIGURE 2:**
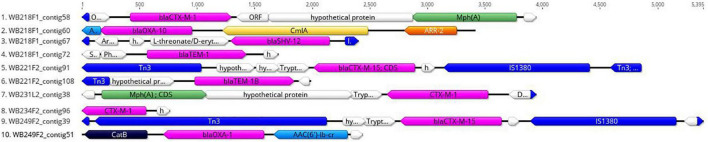
Genomic context of ESβL genes in wild boar isolates. Each line represents a contig harbouring the ESβL determinants, and it is identified by the name of strain and number of the contig. *bla*_*CTX*_, *bla*_*OXA*_, *bla*_*TEM*_, and *bla*_*SHV*_ genes are coloured in purple, while the mobile elements are evidenced in blue. Green, yellow, orange, and light blue indicate other AMR genes, while hypothetical or other proteins are white.

The replicon position at the contig level was compared with the position of ESBL determinants, assuming that, if AMR genes were carried by a plasmid, they would be found near the replicons. The analysis revealed that none of them were located on the same contig of plasmid replicons. In particular, the beta-lactamase genes are located on short contigs (Figure 2), preventing a complete view of the genomic makeup around them. Anyway, the annotation of these contigs was performed and evidenced that *bla*_*TEM–*1*B*_, *bla*_*CTX–M–*1_, and *bla*_*SHV–*12_ genes of WB218F1 strain were surrounded by IS6 and Tn3 family transposase (Figure 2), differently from *bla*_*OXA–*1_, which was not enclosed by any transposable elements. The same situation was detected for other strains. *bla*_*CTX*–M–15_ and *bla*_*TEM–*1*B*_ genes of WB221F2 strain were bracketed with Tn3-such as transposase and TnpA, as well as *bla*_*CTX–M–*15_ of WB249F2; no mobile genetic elements were detected near *bla*_*CTX–M–*1_ gene of WB231L2 and WB234F2 strains. Moreover, the Blastn search evidenced the presence of the transposon ISEcp1 near the *bla*_*CTX–M–*15_ genes in WB231L2 and WB249F2.

### Phylogenetic Insight of Wild Boar ESβL-Producing *Escherichia coli* and the Correlation With ST131 and ST10 ESβL-Producing *Escherichia coli* Isolated From Different Sources

**FIGURE 3:**
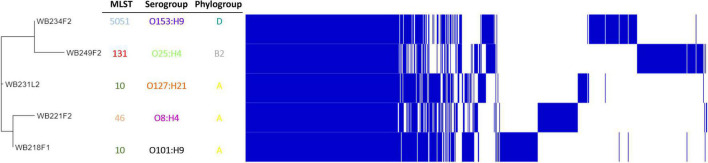
Pangenome analysis of ESβL-producing *Escherichia coli* isolated from wild boars. The figure evidence, on the left, phylogenetic tree, built on SNPs of core genes. Identification of Sequence Type (MLST), serogroup, and phylogroup are also indicated.

To understand how strains were genetically correlated, the pan-genome analysis was carried out. The phylogenetic relationship between wild boar strains is evidenced in Figure 3. All the strains shared 3,325 genes (45% of pangenome), which represent the core genome; the remaining non-core 4,085 genes were divided into 1,316 accessory genes (18% of pangenome) shared by 2–4 strains and 2,769 unique genes (cloud genes; 37% of pangenome). The phylogenetic tree built on SNPs of core genes divided strains into three groups, showing a high degree of heterogeneity inside and reflecting the difference in the genetic composition in terms of virulence, AMR profile, ST, and serogroup.

**FIGURE 4:**
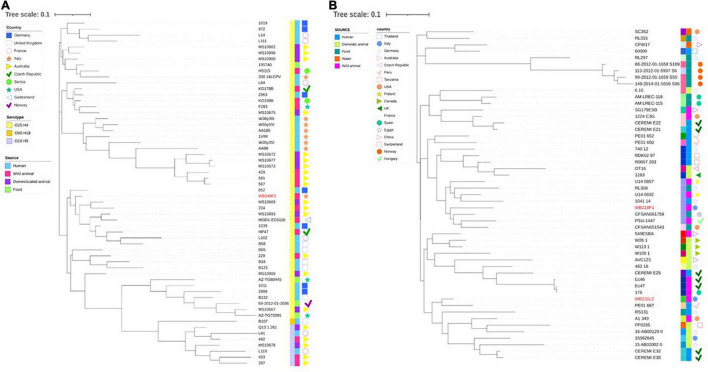
Phylogenetic tree built on the SNPs of core genes in the ST131 **(A)** and ST10 **(B)** ESβL-producing *E. coli* strains analysed. **(A)** WB249F2, the ST131 strain isolated from wild boar, was compared with other 57 strains selected by isolation source and country, and **(B)** the same analysis was performed comparing WB218F1 and WB231L2 with other 49 strains, chosen following the same criteria. The wild boar isolates are highlighted in red in order to better evidence them. Colour-coding is used to report the serotype (first line) and isolation source (second line), while isolation countries are represented by different symbols.

Then, wild boar isolates with ST131 and ST10 were considered for further investigation, with the aim to compare them with other strains with the same ST, isolated from different sources, namely, human, food, wild, and domestic animals. While the phylogenetic tree shows a clear clusterisation of analysed depending on the serotype (Figure 4A), including the wild boar ST131 strain in the O25:H4 group, it also reveals a wide distribution of genomes belonging to different sources and countries. The same situation is observed for the comparison among the ST10 strains (Figure 4B). Although for the ST10 genomes, a more heterogeneous situation was observed regarding the serogroups, the relationship between wild boar isolates and other strains seems not to follow a strict criterion. Interestingly, the two strains are located far from each other, placing WB231L2 near human and domestic animals, while the WB218F1 is near to food and wild animal isolates.

## Discussion

The epidemiology of ESβL-producing microorganisms is quite complicated, including geographical areas, hospitals, communities, hosts, as well as various reservoirs represented by the environment (soil and water), farmed animals, wild animals, and pets. Transmission from food and water, or from direct contact (person-to-person), represents the final step of ESβL-producing bacterial epidemiology (Carattoli, 2008).

The role of wild animals, including wild boars, as maintenance hosts of ESβL-producing *E. coli*, has already been assessed worldwide (Wasyl et al., 2018; Darwich et al., 2021; Palmeira et al., 2021), with some phylogenetic groups showing clinical relevance (Cristóvão et al., 2017). The acquisition of ESβL-producing *E. coli* by wild boars has been proposed to be related to their proximity to human and other animals’ habitats, as well as by their omnivorous habits which may give the animals the possibility to eat human food waste (Poeta et al., 2009), as well as carrions and vegetables contaminated by animal manure.

The major aim of our study was the genomic characterisation of EsβL-producing *E. coli* isolated from wild boars in terms of AMR, virulence, mobile genetic elements, and epidemiology (phylogroups and MLSTs). Since these animals were hunted in mountain areas with limited anthropic influence but can also move in a wider area, they can be considered as indicators of the environmental load of AMR. The comparison with similar studies performed in Northern Italy confirms the high prevalence of ESβL-producing *E. coli*, as 15.96% of ESβL/AmpC *E. coli*-positive wild boars hunted during 2017–2020 in the Lombardy region (Formenti et al., 2021). In our study, where the overall prevalence of ESβL-*E. coli* carriers reached 23.3%, the adults were more frequently positive (35.5%) if compared with the young (26.6%), and the subadults (0.0%), probably because they could move in wider territories and come in contact with different ESβL sources. While young wild boars (0–12 months) were highly exposed to the colonisation due to their impaired immune system, subadults (12–24 months) were probably more reactive in eliminating the microorganisms from the gut.

In this study, all the suspected ESβL-producing *E. coli* showed a MDR profile, with a high percentage (>60%) of fluoroquinolone non-susceptibility level. This represents a particular concern, as this antibiotic class is frequently employed to treat clinical infections. The ESβLs of CTX-M-type resulted in the most spread 3GC/4GC hydrolysing enzymes among the collected isolates. Although this fact is already established and reported in the literature, it is nevertheless interesting to note that the allelic variants identified included the hyper-represented and globally widespread bla_*CTX–M–*1_ and bla_*CTX–M–*15_. We also found the bla_*CTX–M–*245_ variant, neither described earlier in Italy nor in Europe. It was reported only once from a Serratia marcescens isolate collected from a blood sample of a neonate in Iraq (Sahan and Hamed, 2020). This finding suggests that human-animal proximity could also lead to the exchange and circulation of under-detected variants of resistance genes.

The transferability of some resistance plasmids, mainly those ESβL-harbouring, is a further cause for concern. In addition to the ESβL encoding genes, the isolates studied also showed fluoroquinolone and aminoglycoside resistance genes, as *qnrS*, *aac(60)Ibcr*, and *aadA* alone or in combination. Interestingly, the molecular typing showed very high heterogeneity in terms of clonal lineages; isolates collected from different sites (MLNs and faeces) of the same wild boar resulted not clonally related to each other.

Among the strains isolated from wild boars, five were selected in order to be deeper characterised from a genomic point of view. Interestingly, the *in silico* genome analysis highlighted a pandemic clone ST131 isolated from wild boar faeces, belonging to the O25:H4 serogroup and B2 phylogroup. This clone is recognised as a highly virulent group of ExPEC (extraintestinal pathogenic *E. coli*), which is responsible for urinary tract infections, bacteraemia, urinary sepsis, and neonatal sepsis. This strain harbours genomic features typical of ESβLs ExPEC since it shows bla_*CTX–M–*15_ gene, other than bla_*OXA–*1_, and virulence pattern which includes genes responsible for improved adherence and ability to survive in the human body (Sarowska et al., 2019). Since the worldwide diffusion of ST131 clones in humans and also in wildlife (Costa et al., 2006; Wang et al., 2017; Sabença et al., 2021), it is not surprising to find this ST in wild boars (Alonso et al., 2017; Holtmann et al., 2021). This fact is supported by the phylogenetic analysis which places WB249F2 near human isolates and also near wild and domestic animals.

While the ST131 clone commonly shows a rich set of virulence genes (Johnson et al., 2010; Blanco et al., 2011), we found other STs with a less extensive virulence pattern. Notably, we isolated two ST10 strains, which are frequently responsible for AMR-resistant human infection (Day et al., 2016; Pietsch et al., 2017). The same ST was detected in wild boars in Germany (Holtmann et al., 2021), and it is known to be common, especially in poultry (Falgenhauer et al., 2018; Ramos et al., 2020). From the analysis of core genome SNPs, the correlation between ST10 wild boar strains and other strains isolated from different origins, especially human and food, from one side, and human and domestic animal from the other side, has been highlighted. These results confirm again the heterogeneity observed among the isolates of this study, suggesting that wild boars could be a carrier and spreader of any type of ESβL-producing *E. coli* clones.

The other STs detected seem not to be widespread. In fact, the ST46 was found rarely in water in Bangladesh (Rashid et al., 2015) and Chinese companion animals (Chen et al., 2019), while no other authors detected the ST5051 clone.

The capability of AMR genes to be shared with other bacteria in the same niche represents a major threat. This possibility is strictly connected to the genome organisation and genomic feature of *E. coli*, in particular with mobile genetic elements, namely, plasmids, transposons, and insertion sequences. With the aim to investigate if the AMR genes could be transferred to other strains, the genomes of five strains were screened. Overall, the ESβL genes were found to be surrounded by different types of transposons, despite the assembly procedure was not able to reconstruct in a definitive way the genomic context. Since the mobility of AMR can be driven not only by the entire plasmid but also by transposable elements, we cannot exclude the exchange of the AMR gene through this mechanism. Further analysis should be performed using long-read sequencing, as suggested by others (Arredondo-Alonso et al., 2017).

## Conclusion

The main finding of our study is the detection of the ExPEC clone ST131 and the ESβL-MDR ST10 *E. coli* isolates in a restricted wild boar population living in Northern Italy, thus suggesting a circulation of human pathogenic *E. coli* strains among wildlife. Since the concept of One Health is based on the connection between humans, animals, plants, and environmental health, especially in geographically closed systems such as the area of the study, we can assess that wild animals should be included in each AMR surveillance programme. Their role as indicators of the environmental load of AMR is especially evident in our study since all ESβL-producing *E. coli* strains showed an MDR phenotype, including resistance to highly important antimicrobials.

Given the importance of genome analyses to recognise the different *E. coli* clones and trace their occurrence in humans as well as in other species, we strongly suggest the WGS as the most effective tool to investigate ESβL-producing *E. coli*.

## Data Availability Statement

Sequencing data were submitted to the Sequence Read Archive (SRA) under the Project ID PRJNA759409.

## Author Contributions

SB collected samples and performed the strain isolation. AA, AP, AM, FM, and SB performed phenotypic and molecular analysis. CC and GM carried out the bioinformatic analysis. AP, CC, and SB wrote the manuscript. PC, RM, and SB supervised the experiment design and all the analysis and contributed to the manuscript revision. All authors have read and approved the final draft of the manuscript.

## Conflict of Interest

The authors declare that the research was conducted in the absence of any commercial or financial relationships that could be construed as a potential conflict of interest.

## Publisher’s Note

All claims expressed in this article are solely those of the authors and do not necessarily represent those of their affiliated organizations, or those of the publisher, the editors and the reviewers. Any product that may be evaluated in this article, or claim that may be made by its manufacturer, is not guaranteed or endorsed by the publisher.

## Footnotes

1
http://www.eucast.org

2
http://blast.ncbi.nlm.nih.gov/Blast.cgi

3
https://github.com/tseemann/abricate
