# Supplementary material for: Wild Boars as an Indicator of Environmental Spread of ESβL-Producing Escherichia coli
Source: Front Microbiol. 2022 Apr 1;13:838383. doi: 10.3389/fmicb.2022.838383 (PMC9011151; doi:10.3389/fmicb.2022.838383)
Supplement: Supplementary file 1 [file Data_Sheet_1.docx]

Supplementary Material

**Supplementary table S1**. List of ST131 ESβL-producing *E.coli* genomes used for the comparison with WB249F2, the ST131 strain isolated from wild boars. Genomes were selected by country (UK: United Kingdom; US: United States), source (H: *Homo sapiens*; CLF: *Canus lumpus familiaris*; FC: *Felis catus*; F: food; WA: Wild Animal), and ESβL genes variants.

| **Strain** | **Accession number** | **Serotype** | **Country** | **Source** | ***Bla*_tem_** | ***Bla*_ctx_** | ***Bla*_oxa_** | ***Bla*_kpc_** | ***Bla*_cmy_** |
| --- | --- | --- | --- | --- | --- | --- | --- | --- | --- |
| 1011 | GCA_001647335.1 | O25:H4 | Germany | H | blaTEM-235 |  |  |  |  |
| 1019 | GCA_001647315.1 | O25:H4 | Germany | H | blaTEM-1 |  |  |  |  |
| 1039 | GCA_001647325.1 | O25:H4 | Germany | H | blaTEM-1 |  | bla OXA-1 |  |  |
| 2963 | GCA_001561105.1 | O25:H4 | Germany | H | blaTEM-1 | blaCTX-M-27 |  |  |  |
| 2999 | GCA_001561195.1 | O25:H4 | Germany | H | blaTEM-235 |  |  |  |  |
| 852 | GCA_001555495.1 | O25:H4 | Germany | H | blaTEM-1 |  | bla OXA-1 |  |  |
| 972 | GCA_001555635.1 | O25:H4 | Germany | H | blaTEM-1 |  |  |  |  |
| B107 | GCA_001561735.1 | O86:H18 | UK | H | blaTEM-1 |  |  |  |  |
| B125 | GCA_001561785.1 | O25:H4 | UK | H |  | blaCTX-M-15 |  |  |  |
| B132 | GCA_001561705.1 | O25:H4 | UK | H |  | blaCTX-M-1 |  |  |  |
| B16 | GCA_001561275.1 | O25:H4 | UK | H |  | blaCTX-M-15 | bla OXA-1 |  |  |
| B58 | GCA_001561495.1 | O25:H4 | UK | H | blaTEM-1 |  |  |  |  |
| B65 | GCA_001561285.1 | O25:H4 | UK | H | blaTEM-1 | blaCTX-M-15 | bla OXA-1 |  |  |
| L102 | GCA_003427875.1 | O25:H4 | France | H |  |  | bla OXA-1 |  |  |
| L110 | GCA_003427575.1 | O16:H5 | France | H | blaTEM-1 |  |  |  |  |
| L111 | GCA_003427815.1 | O25:H4 | France | H | blaTEM-1 | blaCTX-M-1 |  |  |  |
| L14 | GCA_003428115.1 | O25:H4 | France | H |  | blaCTX-M-1 |  |  |  |
| L41 | GCA_003428105.1 | O16:H5 | France | H | blaTEM-1 | blaCTX-M-1 |  |  |  |
| L64 | GCA_003427895.1 | O25:H4 | France | H | blaTEM-30 |  |  |  |  |
| sk35y35t | ERX4530492 | O25:H4 | Italy | H | blaTEM-90 | blaCTX-M-15 | blaOXA-1 and 9 | blaKPC-2 |  |
| sk36y36t | ERX4530493 | O25:H4 | Italy | H | blaTEM-122 | blaCTX-M-27 | blaOXA-9 | blaKPC-2 |  |
| sk55y55t | ERX4530512 | O25:H4 | Italy | H | blaTEM-79 | blaCTX-M-27 | blaOXA-9 | blaKPC-3 |  |
| 208_14LCPV | data unpublished | O25:H4 | Italy | H | blaTEM-1 |  |  |  |  |
| AA185 | data unpublished | O25:H4 | Italy | H | blaTEM-1 | blaCTX-M-27 | blaOXA-9 | blaKPC-3 |  |
| AA88 | data unpublished | O25:H4 | Italy | H | blaTEM-1 |  | blaOXA-9 | blaKPC-3 |  |
| 1VIM | data unpublished | O25:H4 | Italy | H | blaTEM-1 and 79 |  | blaOXA-9 | blaKPC-2 |  |
| MS10667 | GCA_018728485.1 | O25:H4 | Australia | CLF | blaTEM-1 |  |  |  |  |
| MS10669 | GCA_018728495.1 | O25:H4 | Australia | CLF |  |  | bla OXA-1 |  |  |
| MS10672 | GCA_018728425.1 | O25:H4 | Australia | CLF |  | blaCTX-M-15 | bla OXA-1 |  |  |
| MS10673 | GCA_018728365.1 | O25:H4 | Australia | CLF |  | blaCTX-M-15 | bla OXA-1 |  |  |
| MS10675 | GCA_018728385.1 | O25:H4 | Australia | FC |  | blaCTX-M-27 |  |  |  |
| MS10677 | GCA_018728345.1 | O25:H4 | Australia | CLF |  | blaCTX-M-15 | bla OXA-1 |  |  |
| MS10678 | GCA_018728325.1 | O16:H5 | Australia | FC |  | blaCTX-M-14 |  |  |  |
| MS10893 | GCA_018728245.1 | O25:H4 | Australia | CLF | blaTEM-1 | blaCTX-M-15 |  |  |  |
| MS10898 | GCA_018728265.1 | O25:H4 | Australia | CLF | blaTEM-1 |  |  |  |  |
| MS10900 | GCA_018728225.1 | O25:H4 | Australia | CLF | blaTEM-1 |  |  |  |  |
| MS10902 | GCA_018728155.1 | O25:H4 | Australia | CLF | blaTEM-1 | bla CTX-M-14 |  |  |  |
| MS10908 | GCA_018728145.1 | O25:H4 | Australia | CLF | blaTEM-1 |  |  |  |  |
| Q13_1_261 | GCA_018728525.1 | O16:H5 | Australia | CLF | blaTEM-1 |  |  |  |  |
| 591 | SRR7724635 | O25:H4 | Australia | WA |  | blaCTX-M-15 | blaOXA-1 |  |  |
| 567 | SRR7724756 | O25:H4 | Australia | WA |  | blaCTX-M-15 | blaOXA-1 |  |  |
| 429 | SRR7724649 | O25:H4 | Australia | WA |  | blaCTX-M-15 | blaOXA-1 |  |  |
| 442 | SRR7724742 | O16:H5 | Australia | WA | blaTEM-1 | blaCTX-M-15 |  |  |  |
| 433 | SRR7724657 | O16:H5 | Australia | WA |  | blaCTX-M-15 |  |  |  |
| 297 | SRR7724771 | O16:H5 | Australia | WA |  | blaCTX-M-15 |  |  |  |
| 229 | SRR7724629 | O25:H4 | Australia | WA |  | blaCTX-M-15 | blaOXA-1 |  |  |
| 224 | SRR7724623 | O25:H4 | Australia | WA | blaTEM-1 | blaCTX-M-15 |  |  |  |
| HP47 | GCA_001561865.1 | O25:H4 | Czech Republic | WA | blaTEM-1 | blaCTX-M-15 | blaOXA-1 |  |  |
| KO178B | GCA_001637865.1 | O25:H4 | Czech Republic | WA |  | blaCTX-M-27 |  |  |  |
| KO198B | GCA_001638005.1 | O25:H4 | Serbia | WA |  | blaCTX-M-27 |  |  |  |
| F283 | GCA_001561845.1 | O25:H4 | USA | WA |  | blaCTX-M-27 |  |  |  |
| HS115 | GCA_001561895.1 | O25:H4 | Serbia | WA | blaTEM-1 | blaCTX-M-15 | blaOXA-1 |  |  |
| MOD1-EC6116 | GCA_002463555.1 | O25:H4 | Switzerland | WA | blaTEM-1 | blaCTX-M-15 |  |  |  |
| AZ-TG73391 | SRR1220791 | O25:H4 | USA | F | blaTEM-1 |  |  |  |  |
| AZ-TG60445 | SRR1178255 | O25:H4 | USA | F | blaTEM-1 |  |  |  |  |
| 69-2012-01-3586 | ERR4769384 | O25:H4 | Norway | F |  |  |  |  | blaCMY-2 |
| 195740 | SRR5031226 | O25:H4 | UK | F | blaTEM-1 |  |  |  |  |

**Supplementary table S2**. List of T10 ESβL-producing *E.coli* genomes used for the comparison with WB218 and WB231, the ST10 strain isolated from wild boars. Genomes were selected by country (UK: United Kingdom; US: United States), source (H: *Human*; DA: domestic animal; F: food; WA: Wild Animal; W: water), and ESβL genes.

| **STRAIN** | **ACCESSION NUMBER** | **serotype** | **country** | **source** | **bla-TEM** | **bla-CTX** | **bla-OXA** | **bla-SHV** | **bla-CMY** | **blaNDM** |
| --- | --- | --- | --- | --- | --- | --- | --- | --- | --- | --- |
| 60000 | GCA_014117545.1 | O nd: H12 | Thailand | H | blaTEM-1 | blaCTX-M-3 | blaOXA-1 | blaSHV-12 |  | blaNDM-1 |
| 15062645 | GCA_018193775.1 | O117:H10 | Italy | DA | blaTEM-150 | blaCTX-M-1 |  |  |  |  |
| 1041_14 | GCA_014691585.1 | O101:H9 | Germany | H | blaTEM-154 |  |  |  | blaCMY-2 |  |
| 15_AB02002_0 | PRJEB21546 | O8:H32 | Germany | DA | blaTEM-150 |  |  |  |  |  |
| 16-AB00129_0 | PRJEB21546 | O50:H27 | Germany | DA |  | blaCTX-M-1 |  |  |  |  |
| 482_16 | PRJNA514245 | O132:H28 | Germany | DA | blaTEM-235 |  |  |  | blaCMY-2 |  |
| 6_16 | PRJNA514245 | O nd: H4 | Germany | DA | blaTEM-1 |  |  |  | blaCMY-2 |  |
| 740_12 | GCA_014691485.1 | O101:H10 | Germany | H | blaTEM-1 |  |  |  | blaCMY-2 |  |
| AVC123 | GCA_003338865.1 | O132:H21 | Australia | DA | blaTEM-1 |  |  |  |  |  |
| OT16 | GCA_003591315.1 | O nd:H10 | Czech Republic | DA | blaTEM-34 |  |  |  | blaCMY-2 |  |
| PE01_650 | GCA_013425265.1 | O166:H4 | Peru | H | blaTEM-1 |  |  |  |  |  |
| PE01_652 | GCA_013425145.1 | O99:H33 | Peru | H | blaTEM-1 |  |  |  |  |  |
| PE01_667 | GCA_013424045.1 | H21 | Peru | H | blaTEM-150 |  |  |  |  |  |
| R0007_203 | GCA_900239835.1 | O101:H10 | Tanzania | H | blaTEM-235 |  | blaOXA-1 |  |  |  |
| RDK02_97 | GCA_900239925.1 | O101:H10 | Tanzania | H |  |  | blaOXA-1 |  |  |  |
| RL297 | GCA_014683685.1 | O23:H32 | Germany | F |  |  |  |  | blaCMY-132 |  |
| RL308 | GCA_014688505.1 | O101:H9 | Germany | F |  |  |  |  | blaCMY-2 |  |
| RL315 | GCA_014691445.1 | O2:H2 | Germany | F | blaTEM-1 |  |  |  | blaCMY-2 |  |
| RS131 | GCA_014687265.1 | H27 | Germany | H | blaTEM-1 | blaCTX-M-65 | blaOXA-10 and 1 |  | blaCMY-2 |  |
| SC352 | GCA_003358245.1 | O127:H16 | USA | W | blaTEM-1 |  |  |  |  |  |
| U14_0692 | GCA_016697145.1 | O101:H9 | Polands | WA | blaTEM-154 |  |  |  |  |  |
| U14_0857 | GCA_016697225.1 | O101:H9 | Polands | WA | blaTEM-1 |  |  |  |  |  |
| W109_1 | GCA_011600965.1 | O50:H4 | Canada | DA |  | blaCTX-M-1 |  |  |  |  |
| W113_1 | GCA_011601165.1 | O28ac/O42:H37 | Canada | DA |  | blaCTX-M-1 |  |  |  |  |
| W26_1 | GCA_011600865.1 | O109:H32 | Canada | DA |  | blaCTX-M-1 |  |  |  |  |
| 1283 | GCA_002310735.1 | O101:H10 | UK | DA |  |  |  |  | blaCMY-2 |  |
| CEREMI_E21 | SAMEA4853120 | O73:H31 | France | H |  |  |  |  | blaCMY-138 |  |
| CEREMI_E22 | SAMEA4853121 | O8:H17 | France | H |  |  |  |  | blaCMY-138 |  |
| CEREMI_E25 | SAMEA4853122 | O71:H48 | France | H | blaTEM-1 | blaCTX-M-1 |  |  |  |  |
| CEREMI_E30 | SAMEA4853123 | O73:H31 | France | H |  | blaCTX-M-1 |  |  |  |  |
| CEREMI_E32 | SAMEA4853124 | O73:H31 | France | H |  | blaCTX-M-1 |  |  | blaCMY-138 |  |
| 176 | GCA_012618795.1 | O16:H48 | Spain | WA | blaTEM-122 |  |  |  |  |  |
| 1224_C3G | GCA_014689345.1 | O8:H17 | USA | WA |  |  |  | blaSHV-12 |  |  |
| 549ESBA | GCA_014158385.1 | O26:H32 | Australia | WA | blaTEM-1B | blaCTX-M-15 |  |  |  |  |
| AM_LREC-115 | GCA_017783665.1 | O153:H10 | Spain | WA | blaTEM-1A | blaCTX-M-32 |  |  |  |  |
| CFSAN061759 | PRJNA230969 | O101:H9 | Egypt | WA | blaTEM-1B | blaCTX-M-15 |  |  |  |  |
| CPW17 | PRJNA408214 | O86:H12 | China | F | blaTEM-1B | blaCTX-M-14 | blaOXA-1 |  |  |  |
| Ec47 | PRJNA705836 | O16:H48 | France | F | blaTEM-122 |  |  |  |  |  |
| PF9285 | GCA_004771235.1 | O117:H4 | Switzerland | W | blaTEM-1B |  |  |  |  |  |
| CFSAN051543 | GCA_012664925.1 | O9:H9 | USA | WA | blaTEM-1B |  |  |  |  |  |
| AM_LREC-118 | GCA_017783505.1 | O153:H10 | Spain | DA | blaTEM-1A | blaCTX-M-32 |  |  |  |  |
| 113-2012-01-5997_S6 | SAMEA7483614 | O125:H4 | Norway | F |  |  |  |  | blaCMY-2 |  |
| 88-2012-01-1658_S189 | SAMEA7483587 | O nd:H4 | Norway | F |  |  |  |  |  |  |
| 99-2012-01-1659_S50 | SAMEA7483588 | O nd:H4 | Norway | F |  |  |  |  |  |  |
| 148-2014-01-5656_S86 | SAMEA7483649 | O nd:H4 | Norway | F |  |  |  |  |  |  |
| A1_349 | SAMN14534474 | O45:H45 | USA | F |  | blaCMY-2 |  |  |  |  |
| Ec46 | SAMN18104154 | O16:H48 | France | F | blaTEM-1B |  |  |  |  |  |
| PSU-1447 | SAMN10315796 | O101:H9 | Hungary | WA | blaTEM-1A |  |  |  |  |  |
| SG179ESB | GCA_014779015.1 | O92:H33 | Australia | WA |  | blaCTX-M-15 |  |  |  |  |
